# Arbaclofen extended-release tablets for spasticity in multiple sclerosis: randomized, controlled clinical trial

**DOI:** 10.1093/braincomms/fcac300

**Published:** 2022-11-23

**Authors:** Darin T Okuda, Daniel Kantor, Mark Jaros, Tina deVries, Samuel Hunter

**Affiliations:** Neurology, The University of Texas Southwestern Medical Center, Dallas, TX 75390-8806, USA; Neurology, Florida Atlantic University, Boca Raton, FL 33431, USA; Clinical Immunology, Nova Southeastern University, Fort Lauderdale, FL 33314, USA; Summit Analytical, Denver, CO 80238, USA; Research and Development, RVL Pharmaceuticals, Inc., Bridgewater, NJ 08807, USA; Advanced Neurosciences Institute, Franklin, TN 37064, USA

**Keywords:** spasticity, multiple sclerosis, arbaclofen, extended-release, baclofen

## Abstract

Baclofen, a racemic GABA-B (GABA_B_) receptor agonist, is commonly used for the management of multiple sclerosis-related spasticity but is associated with frequent dosing and poor tolerability. Arbaclofen, the active R-enantiomer of baclofen, exhibits 100- to 1000-fold greater specificity for the GABA_B_ receptor compared with the S-enantiomer and ∼5-fold greater potency compared with racemic baclofen. Arbaclofen extended-release tablets have a dosing interval of 12 hours and have shown a favourable safety and efficacy profile in early-phase clinical development. The current Phase 3 study was designed to evaluate the efficacy and safety of arbaclofen extended-release tablets in patients with multiple sclerosis-related spasticity. In this multicentre, double-blind, placebo-controlled study, adults with multiple sclerosis-related spasticity were randomized to arbaclofen extended-release 40 mg/day, arbaclofen extended-release 80 mg/day or placebo for 12 weeks. The co-primary end-points were the change from baseline to Week 12 in the Total Numeric-transformed Modified Ashworth Scale in the Most Affected Limb score and the Clinical Global Impression of Change score. A hierarchical testing procedure was used to evaluate the co-primary end-points; analyses for the 80 mg/day group were considered inferential only if the arbaclofen extended-release 40 mg/day and placebo groups demonstrated a statistically significant difference (*P* ≤ 0.05) for both end-points. Five hundred thirty-six patients were included in the study. At Week 12, the least squares mean change from baseline in Total Numeric-transformed Modified Ashworth Scale in the Most Affected Limb score was −1.67 (95% confidence interval: −1.97 to −1.36) and −1.28 (95% confidence interval: −1.57 to −0.99) in the arbaclofen extended-release 40 mg/day and placebo groups, respectively (least squares mean difference: −0.39; *P* < 0.048). Improvements were seen in the mean Clinical Global Impression of Change scores for both the arbaclofen extended-release 40 mg/day and placebo groups; however, no statistically significant difference was observed between them (least squares mean difference: −0.10; *P* = 0.43). Most adverse events were of mild-moderate severity. Arbaclofen extended-release 40 mg/day for 12 weeks significantly reduced multiple sclerosis-related spasticity compared with placebo and was safe and well tolerated over the 12-week treatment period. Although arbaclofen extended-release 40 mg/day improved Clinical Global Impression of Change scores, a significant difference from placebo was not observed.

## Teaser

This 12-week multicenter, double-blind, placebo-controlled study reported by Okuda *et al*. assessed the efficacy and safety of arbaclofen extended-release tablets in adults with multiple sclerosis-related spasticity. Arbaclofen extended-release 40 mg significantly improved spasticity symptoms and numerically improved clinicians’ impressions of patient improvement. Most adverse events were of mild-to-moderate severity.

## Graphical Abstract

**Graphical abstract:**
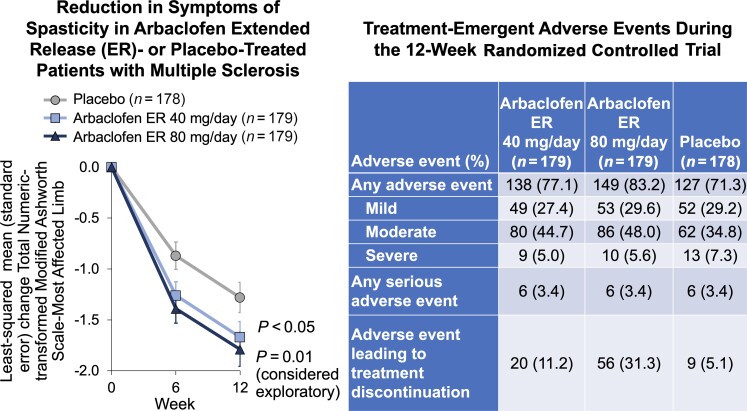

## Introduction

Spasticity affects as many as 80% of patients with multiple sclerosis, an idiopathic inflammatory demyelinating disease of the CNS.^1–4^ Spasticity is characterized by involuntary contractions or shortening of muscle tissues caused by impaired transmission of inhibitory impulses via nerve fibres in the CNS.^5^ One third of patients with multiple sclerosis experience moderate, severe or total limitation in physical ability as a result of spasticity, which can be associated with pain, fatigue, gait disturbance, impaired sleep and bladder dysfunction.^1,5,6^

A primary goal of comprehensive care in multiple sclerosis involves the management of spasticity symptoms. The most commonly prescribed treatment for multiple sclerosis-related spasticity is baclofen, a racemic GABA-B (GABA_B_) receptor agonist that inhibits excitatory signal transmission via supraspinal and spinal cord synapses.^2,7^ Patients with multiple sclerosis treated with baclofen have shown improvements in various measures of spasticity in placebo-controlled clinical studies.^8–10^ However, baclofen has been associated with poor transport across the blood–brain barrier and poor tolerability owing to adverse events, such as sedation, drowsiness and worsening fatigue.^1,7,11,12^ Baclofen is administered in divided doses up to four times daily, increasing the potential for suboptimal adherence, suboptimal dosing and limited therapeutic benefit.^1,5,7,11^

Evidence from *in vitro* and animal studies suggests that the therapeutic effect of racemic baclofen is primarily attributable to the active R-enantiomer, arbaclofen.^13–16^ Arbaclofen exhibits 100- to 1000-fold greater specificity for the GABA_B_ receptor compared with the S-enantiomer and ∼5-fold greater potency compared with racemic baclofen.^15,17–20^ Arbaclofen extended-release (ER) employs osmotic pump technology to provide controlled release and sustained concentrations of the drug, allowing a dosing interval of 12 hours. The pharmacokinetics and pharmacodynamic effects of arbaclofen ER differ from those of racemic baclofen preparations. Consequently, there is neither a fixed weight nor a molar equivalence between arbaclofen ER and the R-enantiomer in racemic baclofen proportional to the drug concentration or to its effect. Although this is principally due to bioavailability differences between these compounds, other factors involving interactions between enantiomers are also plausible. In Phase 1 studies, administration of arbaclofen ER to healthy volunteers at doses ranging from 10–80 mg/day for up to 20 days was generally well tolerated (data on file, RVL Pharmaceuticals, 2017).

The Phase 3 clinical trial programme for arbaclofen ER tablets included two randomized controlled trials and two long-term open-label studies in subjects with spasticity related to multiple sclerosis. In a multicentre, double-blind, parallel-group study of arbaclofen ER (40 mg/day, *n* = 110), baclofen (80 mg/day, *n* = 113) and placebo (*n* = 118), twice-daily arbaclofen ER was efficacious and well tolerated (ClinicalTrials.gov, NCT01743651).^21^ Both arbaclofen ER and baclofen demonstrated improvement in spasticity. Fewer treatment-emergent adverse events were reported for arbaclofen ER in comparison to baclofen. Here, we report the results of a second Phase 3, multicentre, double-blind, randomized, placebo-controlled trial evaluating the efficacy, safety and tolerability of arbaclofen ER in subjects with multiple sclerosis-related spasticity.

## Materials and methods

### Study design and patient population

The double-blind, randomized, placebo-controlled trial was conducted at 82 sites in 10 countries (USA, Russia, Belarus, Serbia, Bosnia and Herzegovina, Croatia, Bulgaria, Moldova, Poland and Ukraine). Eligible subjects were randomized (1:1:1) to receive arbaclofen ER 40 mg/day, arbaclofen ER 80 mg/day or placebo, administered orally twice daily (arbaclofen ER 20 mg × 2, arbaclofen ER 40 mg × 2 or placebo, respectively) for 12 weeks. Doses were titrated over 9 days to the target dose for the assigned treatment group (Supplementary Figure 1, online supplement). Investigators, evaluators and subjects remained blinded to treatment throughout the course of the study. Subjects completed study visits at baseline and on Days 10, 42 and 84. After completion of the Day 84 study visit, the study drug was tapered over 7 days prior to the final safety evaluation on Day 92. Subjects who completed the randomized, placebo-controlled trial were eligible to participate in a 1-year open-label study (ClinicalTrials.gov, NCT03319732).

A complete list of inclusion and exclusion criteria is contained within the online supplement (Supplementary Methods, online supplement). Eligible subjects were adults (age: 18–65 years) with an established diagnosis of multiple sclerosis according to McDonald criteria,^22^ a documented history of spasticity for at least 6 months prior to screening, a Total Numeric-transformed Modified Ashworth Scale (TNmAS) score ≥2 in the most-affected limb (MAL) and an Expanded Disability Status Scale (EDSS) score ≥3. Subjects taking medications indicated for the treatment of spasticity were required to complete a washout period of up to 21 days prior to randomization. Exclusion criteria included the use of high-dose oral or intravenous methylprednisolone, or equivalent, within 3 months of baseline measures, as well as the use of concomitant medications that might potentially interfere with arbaclofen ER or outcome variables. Concomitant treatment with multiple sclerosis disease-modifying medications was permitted, provided there was no change in dose for at least 3 months prior to screening and the subject was willing to maintain the dose for the duration of the study.

This study was conducted according to the principles set forth in the Declaration of Helsinki. The protocol was approved by the institutional review board or ethics committee at each participating study site. All subjects provided written informed consent prior to enrolment.

### Randomization and masking

Treatment group assignment followed a predetermined list of randomization numbers; each successive number assigned the subject to one of the three groups in random order. Randomization was performed by authorized site personnel using an interactive response system, who assigned a kit number that corresponded to the randomization schedule.

### Study outcomes

The co-primary end-points were the change from baseline to Week 12 in the TNmAS-MAL score and the Clinical Global Impression of Change (CGIC) score at Week 12. The TNmAS is a validated 6-point rating scale evaluating the degree of abnormality in muscle tone or the resistance to passive movements; higher scores indicate more severe spasticity.^23,24^ The TNmAS score was calculated by the evaluator by measuring the modified Ashworth score at each of the following joints bilaterally: shoulder, elbow, wrist, hip, knee and ankle. The sum of the scores for each limb’s three joints was used to determine the MAL. The total limb (TL) score for the entire exam of 12 joints was also calculated. Assessments were performed by a trained evaluator who was not the investigator and who was blinded to treatment assignment and all clinical, laboratory and safety assessments. The CGIC is a global rating scale that quantifies the investigator’s global impression of the change from baseline in the patient’s condition. Scores range from −3 (significant worsening) to +3 (significant improvement), with zero indicating no change.^25^ Investigators had access to all patient data, including the TNmAS-MAL data, and were instructed to rate the change compared with baseline in overall global functional performance as the patients appeared on the day of examination. The clinician was instructed to make a judgment about the total picture of the subject at each visit: the illness severity, the subject’s level of distress and other aspects of impairment and the impact of the illness on functioning. The CGIC is rated without regard to the clinician’s belief that any clinical changes are or are not due to medication and without consideration of the aetiology of the symptoms. Opinions were not limited to the level of spasticity the patient was experiencing.

The pre-specified secondary outcomes included the Patient Global Impression of Change (PGIC) score at the final study visit (Week 13, after dose taper completed), the change from baseline to Week 13 in the EDSS score^26^ and the change from baseline to Week 12 in the TNmAS-TL score. The PGIC is a seven-point scale that measures the change in activity limitations, symptoms and quality of life; higher scores indicate greater overall improvement. The EDSS quantifies multiple sclerosis-related disability on a scale ranging from 0 (normal neurological exam) to 10 (death due to multiple sclerosis).

Safety assessments included adverse events, vital signs, clinical laboratory tests, 12-lead electrocardiograms, the Urinary Symptom Profile (USP) Questionnaire^27^ and the Columbia–Suicide Severity Rating Scale.^28^

### Statistical analysis

The estimated sample size of 151 patients per treatment arm provided 90% power to detect a 0.9-point difference between the arbaclofen ER 40 mg/day and placebo groups in the mean change from baseline to Week 12 in the TNmAS-MAL score and 99% power to detect a 0.5-point difference in the CGIC score at Week 12 based on a two-sided *t*-test and a Type 1 error of 0.05.

The co-primary end-points were analysed using a restricted maximum likelihood-based mixed model with repeated measures (MMRM) and fixed effects for study treatment, visit, country and treatment-by-visit interaction. For the change in the TNmAS score, the baseline score was included as a covariate in the model. Between-group differences in the least squares (LS) mean values for each end-point were tested using a two-tailed *P* value of 0.05. A sequential testing procedure was used to evaluate the co-primary end-points. The primary analysis compared the arbaclofen ER 40 mg/day and placebo groups; *P* values for comparisons between the arbaclofen ER 80 mg/day and placebo groups were considered inferential only if the results of the comparison between the arbaclofen ER 40 mg/day and placebo groups were statistically significant (*P* ≤ 0.05) for both end-points. A statistically significant finding on both co-primary end-points was required to reject the null hypothesis. Pre-specified sensitivity analyses were performed to assess the robustness of the results of the primary analyses and to test the assumptions of the analytic model. Observed cases, without imputation, were used for the majority of analyses. Sensitivity analysis for the co-primary efficacy end-points utilized a modified worst-observation-carried-forward imputation in which missing data were imputed using the worst observation carried forward for subjects who withdrew early due to an adverse event; all other missing data were imputed using the last observation carried forward. A placebo-based pattern mixture model was used to assess the ‘missing at random’ assumptions of the analytical model for both TNmAS-MAL and CGIC.

Secondary outcomes were analysed using the same MMRM that was used in the primary analyses. For the change from baseline in the TNmAS-TL and the EDSS scores, the baseline score was included as a covariate in the model. No adjustments for multiple comparisons were made to the secondary outcomes.

All efficacy analyses were conducted in the intent-to-treat population. Analyses were performed using SAS® software version 9.4 (SAS Institute, Cary, NC). Adverse events were coded according to preferred terms in the Medical Dictionary for Regulatory Activities, version 20.1.^29^ Safety outcomes are reported as events occurring between the first dose of study treatment and 30 days after the last dose of study treatment. A data safety monitoring board comprising four experienced multiple sclerosis clinicians prospectively reviewed comprehensive unblinded safety datasets, including but not limited to clinical laboratory data and adverse events, throughout the duration of the study.

## Results

This study was conducted between January and December 2018. A total of 536 of the 594 subjects screened were enrolled and randomly assigned to receive arbaclofen ER 40 mg/day (*n* = 179), arbaclofen ER 80 mg/day (*n* = 179) or placebo (*n* = 178). Demographics and baseline characteristics were generally well-balanced across treatment groups (Table 1). The mean (standard deviation [SD]) age was 46.5 (9.6) years. Most subjects were Caucasian (97.4%). At baseline, most subjects had a diagnosis of relapsing-remitting multiple sclerosis (60.3%), followed by secondary-progressive multiple sclerosis (34.7%) and primary-progressive multiple sclerosis (5.0%).

**Table 1:** Demographics and baseline characteristics

Characteristic	Arbaclofen ER	Arbaclofen ER	Placebo (*n* = 178)	Total (*N* = 536)
	40 mg/day (*n* = 179)	80 mg/day (*n* = 179)		
Age, mean (SD), years	46.0 (9.50)	46.0 (9.71)	47.5 (9.55)	46.5 (9.60)
Sex, *n* (%)				
Female	108 (60.3)	101 (56.4)	110 (61.8)	319 (59.5)
Male	71 (39.7)	78 (46.3)	68 (38.2)	217 (40.5)
Race, *n* (%)				
Asian	0	0	1 (0.6)	1 (0.2)
Black or African-American	4 (2.2)	0	2 (1.1)	6 (1.1)
Caucasian	171 (95.5)	177 (98.9)	174 (97.8)	522 (97.4)
Mixed	1 (0.6)	0	1 (0.6)	2 (0.4)
Not collected	3 (1.7)	2 (1.1)	0	5 (0.9)
Height, mean (SD), cm	168.9 (8.84)	169.8 (9.45)	169.2 (9.30)	169.3 (9.19)
Weight, mean (SD), kg	70.4 (15.37)	71.5 (15.41)	70.7 (14.83)	70.9 (15.18)
Body mass index, mean (SD), kg/m^2^	24.7 (5.12)	24.7 (4.26)	24.6 (4.40)	24.6 (4.60)
Multiple sclerosis subtype, *n* (%)				
Relapsing-remitting	117 (65.4)	100 (55.9)	106 (59.6)	323 (60.3)
Primary-progressive	9 (5.0)	8 (4.5)	10 (5.6)	27 (5.0)
Secondary-progressive	53 (29.6)	71 (39.7)	62 (34.8)	186 (34.7)
TNmAS-MAL score, mean (SD)	7.4 (3.24)	7.6 (3.02)	7.6 (3.13)	7.5 (3.12)
Prior therapy, *n* (%)				
Baclofen	38 (21.2)	40 (22.3)	36 (20.2)	114 (21.3)
Diazepam	2 (1.1)	1 (0.6)	6 (3.4)	9 (1.7)
Gabapentin	5 (2.8)	0	2 (1.1)	7 (1.3)
Tizanidine	11 (6.1)	16 (8.9)	13 (7.3)	40 (7.5)

A total of 403 (75.2%) subjects completed the study (Supplementary Figure 2, online supplement). Study treatment was discontinued early in 42 (23.5%) and 72 (40.2%) subjects in the arbaclofen ER 40 mg/day and 80 mg/day groups, respectively, compared with 19 (10.7%) in the placebo group. Reasons for early discontinuation included adverse events (12.3%, 31.8% and 6.2% for arbaclofen ER 40 mg/day, 80 mg/day and placebo groups, respectively), subject request (10.1%, 7.3% and 4.5% for arbaclofen ER 40 mg/day, 80 mg/day and placebo groups, respectively), multiple sclerosis relapse (1.1% and 0.6%, for arbaclofen ER 40 mg/day and 80 mg/day groups, respectively) and other reasons (0.6%, for the arbaclofen ER 80 mg/day group).

### Primary efficacy analysis

**Figure 1:**
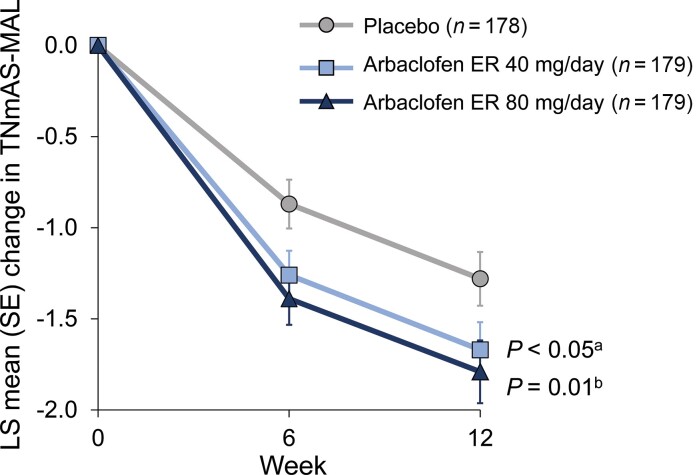
**Least squares mean change from baseline in the TNmAS-MAL score.** The primary outcome, the Total Numeric-transformed Modified Ashworth Scale in the Most Affected Limb (TNmAS-MAL), was analysed using a mixed model with repeated measures and fixed effects for study treatment, visit, country and the treatment-by-visit interaction; baseline score was included as a model covariate. ^a^Arbaclofen ER 40 mg/day versus placebo. ^b^Arbaclofen ER 80 mg/day versus placebo; comparison between the arbaclofen ER 80 mg/day and placebo groups was considered exploratory because statistical significance was not achieved for both co-primary end-points in the comparison between the arbaclofen ER 40 mg/day and placebo groups.

The co-primary end-points of change from baseline to Day 84 in TNmAS-MAL scores and CGIC scores were not met per the pre-specified analysis. Treatment with arbaclofen ER 40 mg/day resulted in a significant improvement in the TNmAS-MAL score at Week 12 compared with placebo. The LS mean change from baseline to Week 12 was −1.67 (95% confidence interval [CI]: −1.97 to −1.36) in the arbaclofen ER 40 mg/day group compared with −1.28 (95% CI: −1.57 to −0.99) in the placebo group (LS mean difference, −0.39; *P* = 0.048; Fig. 1). LS mean scores on the CGIC scale at Week 12 were positive (improved from baseline) in the arbaclofen ER 40 mg/day (0.36; 95% CI: 0.17 to 0.54) and placebo (0.45; 95% CI: 0.27 to 0.63) groups, but there was no statistically significant difference between the treatment and placebo study arms (LS mean difference, −0.10; *P* = 0.43; Table 2).

**Table 2:** CGIC scores and secondary outcomes

	Arbaclofen ER	Arbaclofen ER	Placebo (*n* = 178)
	40 mg/day (*n* = 179)	80 mg/day (*n* = 179)	
Co-primary outcome: CGIC			
LS mean score (95% CI)^a,b^	0.36 (0.17 to 0.54)	0.01 (–0.19 to 0.22)	0.45 (0.27 to 0.63)
*P* value (within treatment test)	<0.001	0.90	<0.01
LS mean difference versus placebo (SE)	–0.10 (0.121)	–0.44 (0.126)	NA
*P* value (versus placebo)	0.43	<0.001^c^	
Secondary outcomes			
TNmAS-TL score			
LS mean change from baseline (95% CI)^b^	–3.11 (−3.81 to −2.42)	–3.33 (−4.09 to −2.57)	–2.42 (−3.09 to −1.75)
*P* value (within treatment test)	<0.001	<0.001	<0.001
LS mean difference versus placebo (SE)	−0.70 (0.455)	−0.91 (0.472)	NA
*P* value (versus placebo)	0.13	0.05	NA
EDSS score			
LS mean change from baseline (95% CI)^d^	–0.03 (−0.07 to 0.01)	0.01 (−0.03 to 0.06)	–0.04 (−0.08 to 0.00)
*P* value (within treatment test)	0.09	0.51	0.07
LS mean difference versus placebo (SE)	0.00 (0.025)	0.05 (0.025)	NA
*P* value (versus placebo)	0.89	0.05	NA
PGIC			
LS mean score (95% CI)^d^	2.67 (2.40 to 2.94)	2.46 (2.17 to 2.75)	2.86 (2.59 to 3.13)
*P* value (within treatment test)	<0.001	<0.001	<0.001
LS mean difference versus placebo (SE)	−0.19 (0.175)	−0.40 (0.177)	NA
*P* value (versus placebo)	0.28	0.02	NA

^a^Mixed model with repeated measures and fixed effects for study treatment, visit, country and the treatment-by-visit interaction. ^b^Assessed at Week 12. ^c^Comparison of co-primary outcomes in the arbaclofen ER 80 mg/day and placebo groups was considered exploratory because statistical significance was not achieved for both co-primary end-points in the comparison between the arbaclofen ER 40 mg/day and placebo groups. ^d^Assessed at the final study visit (Week 13). NA, not applicable; SE, standard error.

In accordance with the pre-specified sequential testing procedure, the comparison of the co-primary endpoints in the arbaclofen ER 80 mg/day and placebo groups were considered exploratory. At Week 12, the LS mean change from baseline in the TNmAS-MAL score in the arbaclofen ER 80 mg/day group was −1.79 (95% CI: −2.12 to −1.46; LS mean difference versus placebo, −0.51; *P* = 0.01). The LS mean score on the CGIC scale at Week 12 in the arbaclofen ER 80 mg/day group was 0.01 (95% CI: −0.19 to 0.22), indicating no worsening, but the LS mean difference in comparison with placebo was −0.44 (*P* < 0.001), suggesting CGIC scores were worse than placebo (Table 2).

The results of the pre-specified sensitivity analyses of the co-primary endpoints were generally consistent with the findings from the primary analyses (Supplementary Tables 1–3, online supplement).

### Secondary outcomes

Secondary outcomes are presented in Table 2. Significant improvements were observed in both the treatment groups and the placebo group in the mean change from baseline to Week 12 in the TNmAS-TL score and in the subjects’ assessment of change in overall status as measured by the PGIC. Significant differences were not observed between the arbaclofen ER 40 mg/day group and the placebo group according to the MMRM analysis of TNmAS-TL. While the LS mean PGIC score for the arbaclofen ER 40 mg/day group and the placebo group were similar (LS mean difference −0.19; *P* = 0.2815), the LS mean PGIC score for the arbaclofen ER 80 mg/day group (2.46; 95% CI: 2.17 to 2.75) was significantly different (lower) compared with the placebo group (2.86; 95% CI: 2.59 to 3.13; LS mean difference −0.40; *P* = 0.0233). No significant difference was observed in the mean change in EDSS score between the baseline and the final study visit in any treatment group.

### Safety outcomes

The most common adverse events are summarized in Table 3. Adverse events occurring with a higher frequency in both arbaclofen ER dose groups compared with placebo included muscular weakness (43 [24.0%], 40 [22.3%] and 27 [15.2%] subjects in the arbaclofen ER 40 mg/day, arbaclofen ER 80 mg/day and placebo groups, respectively) and dizziness (27 [15.1%], 37 [20.7%] and 20 [11.2%], respectively). Somnolence was reported by 20 subjects who received arbaclofen ER 40 mg/day (11.2%), 27 subjects who received arbaclofen ER 80 mg/day (15.1%) and 19 subjects who received a placebo (10.7%). Most adverse events were mild to moderate and without significant clinical consequences. Adverse events led to treatment discontinuation in 22 (12.3%) and 57 (31.8%) subjects in the arbaclofen ER 40 mg/day and arbaclofen ER 80 mg/day groups, compared with 11 (6.2%) subjects in the placebo group. The most common adverse events leading to treatment discontinuation were muscular weakness, asthenia and nausea (Supplementary Table 4, online supplement). There were no differences between treatment groups in the incidence of serious adverse events, clinically significant laboratory abnormalities or electrocardiogram findings.

**Table 3:** **Treatment-emergent adverse events** ^a^

Adverse event (%)	Arbaclofen ER	Arbaclofen ER	Placebo (*n* = 178)	Overall (*N* = 536)
	40 mg/day (*n* = 179)	80 mg/day (*n* = 179)		
Any adverse event	148 (82.7)	154 (86.0)	133 (74.7)	435 (81.2)
Mild	52 (29.1)	56 (31.3)	56 (31.5)	164 (30.6)
Moderate	83 (46.4)	87 (48.6)	63 (35.4)	233 (43.5)
Severe	13 (7.3)	11 (6.1)	14 (7.9)	38 (7.1)
Any serious adverse event	7 (3.9)	6 (3.4)	6 (3.4)	19 (3.5)
Adverse event leading to treatment discontinuation	22 (12.3)	57 (31.8)	11 (6.2)	90 (16.8)
Most common adverse events^b^				
Urinary tract disorder	56 (31.3)	57 (31.8)	67 (37.6)	180 (33.6)
Muscular weakness	43 (24.0)	40 (22.3)	27 (15.2)	110 (20.5)
Asthenia	24 (13.4)	37 (20.7)	27 (15.2)	88 (16.4)
Dizziness	27 (15.1)	37 (20.7)	20 (11.2)	82 (15.3)
Nausea	40 (22.3)	30 (16.8)	28 (15.7)	98 (18.3)
Somnolence	20 (11.2)	27 (15.1)	19 (10.7)	66 (12.3)
Vomiting	14 (7.8)	19 (10.6)	16 (9.0)	49 (9.1)
Gait disturbance	2 (1.1)	14 (7.8)	6 (3.4)	22 (4.1)
Fatigue	4 (2.2)	10 (5.6)	7 (3.9)	21 (3.9)
Vertigo	5 (2.8)	9 (5.0)	4 (2.2)	18 (3.4)
Headache	4 (2.2)	6 (3.4)	12 (6.7)	22 (4.1)

^a^ Treatment-emergent adverse events were any adverse events occurring during the period from the first dose of study drug until 30 days after the last dose of study drug. ^b^Includes adverse events occurring with a frequency ≥5% in any treatment group in the randomized trial or ≥5% in the safety population in the open-label study.

Assessment of mean parameter values on the USP Questionnaire showed no clinically relevant changes from baseline to the final study visit and no evidence of worsening in urinary symptoms in the arbaclofen ER groups compared with placebo (Supplementary Table 5, online supplement). There was no clinical evidence of increased suicidal ideation or behaviour in any treatment group, according to scores on the Columbia–Suicide Severity Rating Scale.

## Discussion

In this Phase 3 multicentre, randomized, double-blind, placebo-controlled trial comparing arbaclofen ER with placebo in subjects with multiple sclerosis-related spasticity, administration of arbaclofen ER for 12 weeks significantly reduced spasticity as measured by the TNmAS-MAL. The clinician’s global impression of change, as measured by the CGIC Scale, improved with arbaclofen ER 40 mg/day and placebo but did not change with arbaclofen ER 80 mg/day. The CGIC score at Week 12 for the arbaclofen ER 40 mg/day group was not different, and the arbaclofen ER 80 mg/day score was worse when compared with the placebo score. As such, the co-primary end-point for the study was not met. The majority of adverse events were mild to moderate. These findings add to the previously reported results of a Phase 3 clinical study and an open-label, long-term extension study of arbaclofen ER 40 mg/day.^21,30^

Spasticity resulting from upper motor neuron injury in multiple sclerosis is heterogeneous, resulting in a spectrum of patient experiences from pain to reduced mobility and function throughout the day.^1,5,6^ Current pharmacologic treatment options for spasticity related to multiple sclerosis are limited and no new therapeutic options have been approved in the USA in over 10 years. Response to pharmacologic treatment also varies and the treatment course is often complicated by the emergence of adverse reactions or treatment intolerance prior to reaching the effective dose.^1,7^ Routine movement, the speed at which a limb is moved and reduced ambient temperatures are just a few key factors that influence the degree of spasticity in patients.^5^ Arbaclofen, which has greater specificity of action and a reduced dosing frequency when compared with baclofen, may provide another solution for spasticity management.

The results from this randomized trial highlight the complexities of measures quantifying benefit in reducing muscle spasticity and associated influences on other realms of function that may impact the clinician’s view of the global functioning of the patient. Here, the demonstrated CGIC outcomes should be interpreted in the context of well-described challenges associated with the objective measurement of spasticity in patients with multiple sclerosis, including the broad inter- and intra-patient variability in the clinical manifestation of symptoms  and the lack of a validated instrument that measures all relevant aspects of the condition.^4,5,31^ Evaluating patients at the same time of day as that of the baseline assessment may have also increased the quality of the data acquired by reducing the impact of outside factors that may have affected outcome measures. In addition, because spasticity can mitigate and amplify the effects of other multiple sclerosis-related symptoms, improvements in spasticity may have resulted in a corresponding worsening of other symptoms including motor strength, thereby limiting the perceived effect of treatment on the overall condition.^10,32–34^ This is supported by the observed outcomes related to the arbaclofen ER 80 mg/day dose, whereby spasticity measures were significantly reduced in the setting of worse CGIC outcomes at Week 12 when compared with placebo. Results of this study showed that arbaclofen ER treatment did not worsen overall patient status as assessed by the CGIC, though it did not achieve a significant improvement relative to a placebo.

Our findings are framed by certain limitations. In the absence of a validated instrument that measures all relevant aspects of multiple sclerosis spasticity, clinical efficacy was evaluated using co-primary end-points measured at a single time point during the day and a sequential testing procedure for the two arbaclofen ER dose groups. Though widely used as an assessment tool in psychiatry, the validity of the CGIC may be affected by factors such as the waxing and waning nature of multiple sclerosis, indication-irrelevant changes and dependence on the rater’s memory.^35,36^ A uniform target dose was also used for all participants and the lack of an adaptive dosing design for tapering coupled with the relatively rapid, forced 9-day titration schedule likely impacted both the retention of individuals randomized to arbaclofen ER and the documented treatment effect. Implementing a strategy based on the minimal effective dose may have resulted in a lower rate of adverse reactions and higher CGIC scores. The contribution of other factors, including social determinants of health, the impact of prescribed disease-modifying medications that may influence confirmed disability outcome measures and lack of participation of a large number of non-whites, is also meaningful.

Overall, arbaclofen was well tolerated by patients, and the observed safety data and level of adherence were in keeping with prior investigations.^21,30^ Adverse events reported here and in a second multicentre, randomized, double-blind, placebo-controlled trial were weakness, dizziness, akathisia and somnolence and were generally mild to moderate and without significant clinical consequence. No new or unexpected adverse events or laboratory abnormalities were identified in this study. The anticipated time frame of treatment effect, observed from the separation of the TNmAS-MAL scores, appeared as early as 1–2 weeks in this study. When arbaclofen ER was slowly titrated over a 6-week dose-response period to 40 mg/day in the trial described above, significant differences in TNmAS-MAL scores were demonstrated as soon as 3 weeks compared with placebo.^21^ While comparisons across clinical trials are not possible due to differences in study methodology and trial populations, both trials support the safety and efficacy profile of arbaclofen ER 40 mg/day.

In conclusion, administration of oral arbaclofen ER 40 mg/day for 12 weeks resulted in a significant improvement in multiple sclerosis-related spasticity as measured by the TNmAS and while CGIC scores improved, they were not significantly different from placebo. Considering the well-characterized limitations of current therapies used for symptomatic management and the unmet need for effective treatments with demonstrated long-term tolerability, these data support the potential therapeutic benefit of arbaclofen ER in patients with multiple sclerosis-related spasticity.

## Supplementary Material

fcac300_Supplementary_Data

## Data Availability

The data that support the findings of this study are not publicly available due to confidentiality restrictions.

## Abbreviations

CGIC =: Clinical Global Impression of Change
CI =: confidence interval
EDSS =: Expanded Disability Status Scale
ER =: extended-release
GABA-B =: GABA_B_
LS =: least squares
MAL =: Most Affected Limb
MMRM =: mixed model with repeated measures
PGIC =: Patient Global Impression of Change
SD =: standard deviation
TL =: Total Limb
TNmAS-MAL =: Total Numeric-transformed Modified Ashworth Scale in the Most Affected Limb
USP =: Urinary Symptom Profile
