# Supplementary material for: Arbaclofen extended-release tablets for spasticity in multiple sclerosis: randomized, controlled clinical trial
Source: Brain Commun. 2022 Nov 23;4(6):fcac300. doi: 10.1093/braincomms/fcac300 (PMC9732858; doi:10.1093/braincomms/fcac300)
Supplement: fcac300_Supplementary_Data [file fcac300_supplementary_data.docx]

**Arbaclofen extended-release tablets for spasticity in multiple sclerosis: randomized, controlled clinical trial**

Darin T. Okuda, MD; Daniel Kantor, MD; Mark Jaros, PhD;
Tina deVries, PhD; Samuel Hunter, MD, PhD

**Supplementary materials**

**Supplementary Figure 1** Dose-titration schedule

**Supplementary Methods** Enrollment criteria

**Supplementary Figure 2** Enrollment and disposition of patients

**Supplementary Table 1** MMRM analysis of TNmAS-MAL and CGIC scores in the modified intention-to-treat population

**Supplementary Table 2** MMRM analysis of TNmAS-MAL and CGIC scores in the per-protocol population

**Supplementary Table 3** MMRM analysis of TNmAS-MAL and CGIC scores with pattern mixture model

**Supplementary Table 4** Adverse events leading to treatment discontinuation

**Supplementary Table 5** USP Questionnaire scores

**References**

**Supplementary Figure 1. Dose-titration schedule***

**
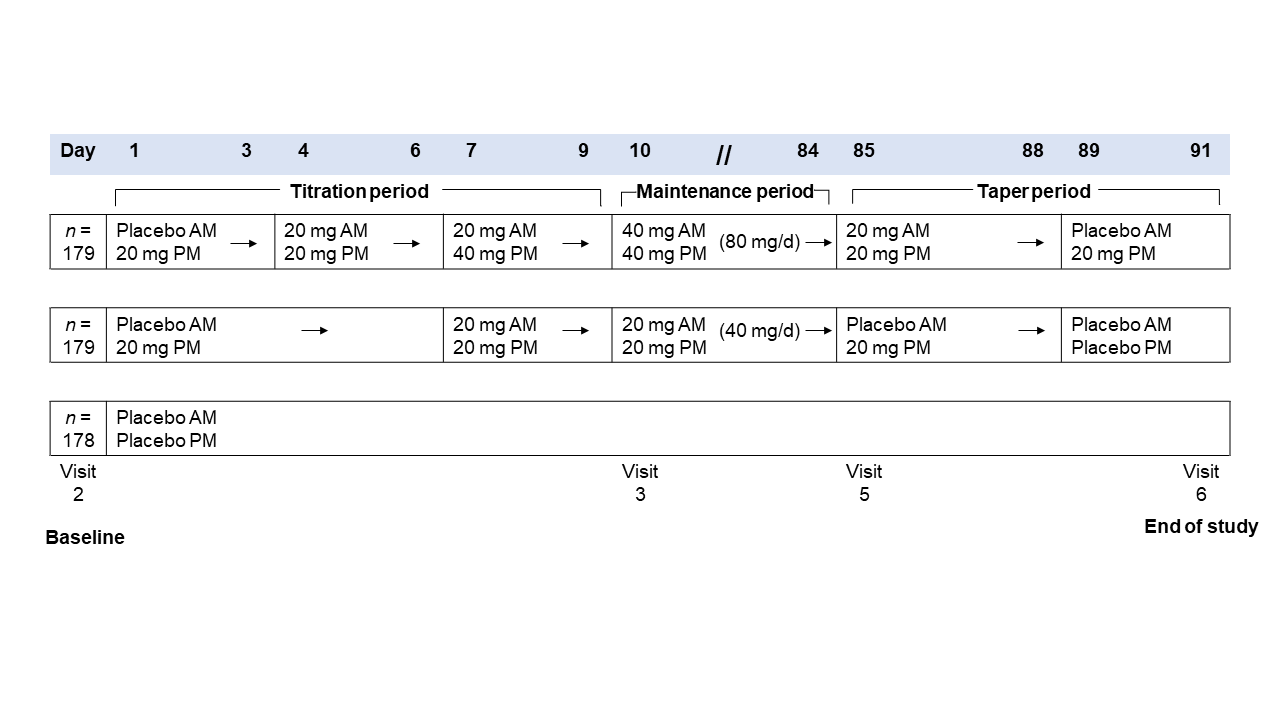
***All doses refer to arbaclofen ER. Each patient received 4 tablets/day, 2 in the morning and 2 in the evening, of either placebo or arbaclofen ER to ensure blinding was maintained during titration and maintenance treatment.

Abbreviations: EOS = end of study; ER = extended-release

**Supplementary Methods. Enrollment criteria**

**Inclusion criteria**

Male and female patients will be considered eligible for participation in the study if all the following inclusion criteria are satisfied at Visit 1 (Screening):

1. Patients 18 to 65 years of age, inclusive.

2. An established diagnosis of multiple sclerosis per McDonald Criteria^1^ (either relapsing-remitting or secondary-progressive course) with a documented history of spasticity for at least 6 months prior to screening.

3. Spasticity due to multiple sclerosis as shown by a Total Numeric-transformed Modified Ashworth Scale (TNmAS) score ≥2 in the most affected limb.

4. Expanded Disability Status Scale (EDSS) score ≥3.0.

5. If receiving disease-modifying medications (eg, interferons approved for multiple sclerosis, glatiramer acetate, natalizumab, fingolimod, or mitoxantrone), there must be no change in dose for at least 3 months prior to Visit 1 (Screening), and the patient must be willing to maintain this treatment dose for the duration of the study. If receiving Ampyra^®^ (dalfampridine, fampridine, 4-amino pyridine), the patient must be on a stable dose for at least 3 months prior to Visit 1 (Screening).

6. Stable regimen for at least 3 months prior to Visit 2 (Baseline) for all medications and non-pharmacological therapies that are intended to alleviate spasticity.

a. Patients taking medications indicated for the treatment of spasticity (eg, baclofen, benzodiazepines, cannabinoids, carisoprodol, dantrolene, tizanidine, cyclobenzaprine, any neuroleptic, ropinirole, tolperisone, and clonidine) at Visit 1 (Screening) must wash out from these medications for at most 21 days by Visit 2 (Baseline) to be eligible for randomization. Patients found not to meet this criterion will be withdrawn from the study and will be considered screen failures.

7. Absence of infections, peripheral vascular disease, painful contractures, advanced arthritis, or other conditions that hinder evaluation of joint movement.

8. Creatinine clearance, as calculated by the glomerular filtration rate (GFR) using the Modification of Diet in Renal Disease Study (MDRD) formula of >50 mL/minute.

9. Use of a medically highly effective form of birth control during the study and for 3 months thereafter for women of child-bearing potential (including female patients and female partners of non-sterile male patients). Use of a medically highly effective form of birth control during the study and for 3 months thereafter for any patient whose partner is not sterilized or post-menopausal.

10. Willing to sign the informed consent form.

**Exclusion Criteria**

Patients who meet any of the following criteria will not qualify for the study.

1. Any concomitant disease or disorder that has symptoms of spasticity or that may influence the patient’s level of spasticity.

2. Inability to rate their level of spasticity or distinguish it from other multiple sclerosis symptoms.

3. Acute multiple sclerosis exacerbation/relapse requiring treatment or disease-modifying drug dose alteration within 3 months of Visit 1 (Screening).

4. Use of high dose (120 mg daily) oral or intravenous methylprednisolone, or equivalent, within 3 months before Visit 1 (Screening).

5. Concomitant use of medications that would potentially interfere with the actions of the study medication or outcome variables.

6. Use of botulinum toxin A or B for spasticity within 6 months of Visit 1 (Screening).

7. Pregnancy, lactation, or planned pregnancy during the course of the study and for 3 months after the final study visit.

8. Recent history (within past 12 months) of any unstable psychiatric disease (or any yes response to questions 1 or 2 on the Columbia–Suicide Severity Rating Scale [C-SSRS] at Screening), or current signs and symptoms of significant medical disorders such as severe, progressive, or uncontrolled pulmonary, cardiac, gastrointestinal, hepatic, renal, genitourinary, hematological, endocrine, immunologic, or neurological disease.

9. History of epilepsy.

10. Current significant cognitive deficit, severe or untreated anxiety, severe or untreated depression.

11. Patients with abnormal micturition that requires indwelling or intermittent catheterization or with lower urinary tract symptoms that result in a score >26 on the Visit 2 (Baseline) Urinary Symptom Profile – (USP^©^) Questionnaire. Patients who are proficient in self-catheterization may be included in the study at investigator discretion.

12. Clinically significant abnormal laboratory values, in the opinion of the investigator, at Visit 1 (Screening).

13. Current malignancy or history of malignancy that has not been in remission for more than 5 years, except effectively treated basal cell skin carcinoma.

14. Any other significant disease, disorder, or significant laboratory finding which, in the opinion of the investigator, puts the patient at risk because of participation, influences the result of the study, or affects the patient’s ability to participate.

15. Planned elective surgery or other procedures requiring general anesthesia during the course of the study.

16. History of any illicit substance abuse (eg, alcohol, cocaine) or prescription for long-acting opioids within the past 12 months (tramadol use will be allowed).

17. Participation in another clinical research study within 1 month of Visit 1 (Screening).

**Supplementary Figure 2.** **Enrollment and disposition of patients**

Assessed for eligibility (n=594)

Discontinued study (n=19)

Adverse event (n=11)^b^

Patient request (n=8)

Randomized (n=536)

Excluded (n=58)

Did not meet eligibility criteria (n=39)

Other reasons^a^ (n=19)

Arbaclofen ER 80 mg/day (n=179)

Received study drug (n=179)

Did not receive study drug (n=0)

Discontinued study (n=42)

Adverse event (n=22)^b^

Patient request (n=18)

MS relapse (n=2)

Arbaclofen ER 40 mg/day (n=179)

Received study drug (n=179)

Did not receive study drug (n=0)

Completed randomized trial (n=137)

Placebo (n=178)

Received study drug (n=178)

Did not receive study drug (n=0)

Discontinued study (n=72)

Adverse event (n=57)^b^

Patient request (n=13)

MS relapse (n=1)

Other (n=1)

^a^Patients were screened but did not return for further assessment.

^b^Includes both treatment-emergent and non–treatment-emergent adverse events.

Abbreviations: ER = extended-release; MS = multiple sclerosis

Completed randomized trial (n=107)

Completed randomized trial (n=159)

**Supplementary Table 1. MMRM analysis of TNmAS-MAL and CGIC scores in the modified intention-to-treat population^a^**

|  | Arbaclofen ER  40 mg/day  (n=177) | Arbaclofen ER  80 mg/day  (n=178) | Placebo  (n=178) |
| --- | --- | --- | --- |
| **TNmAS–MAL score (Week 12)** | | | |
| LS mean change from baseline (95% CI) | –1.66 (–1.96, –1.35) | –1.79 (–2.12, –1.46) | –1.27 (–1.56, –0.98) |
| LS mean difference vs. placebo (SE) | –0.38 (0.196) | –0.52 (0.203) | NA |
| *P* value | <0.05 | 0.01 |  |
| **CGIC (Week 12)** | | | |
| LS mean score (95% CI) | 0.35 (0.16, 0.54) | 0.01 (–0.20, 0.22) | 0.45 (0.27, 0.64) |
| LS mean difference vs. placebo (SE) | –0.10 (0.121) | –0.44 (0.126) | NA |
| *P* value | 0.40 | <0.001 |  |

^a^Modified intention-to-treat population defined as the intention-to-treat population, excluding patients with an MS relapse.

Abbreviations: CGIC = Clinical Global Impression of Change; CI = confidence interval; ER = extended-release; LS = least squares; MMRM = mixed model with repeated measures; MS = multiple sclerosis; NA = not applicable; SE = standard error; TNmAS-MAL = Total Numeric-modified Ashworth Scale-Most Affected Limb

**Supplementary Table 2. MMRM analysis of TNmAS-MAL and CGIC scores in the per-protocol population^a^**

|  | Arbaclofen ER  40 mg/day  (n=175) | Arbaclofen ER  80 mg/day  (n=175) | Placebo  (n=169) |
| --- | --- | --- | --- |
| **TNmAS-MAL score (Week 12)** | | | |
| LS mean change from baseline (95% CI) | ‒1.67 (‒1.97, ‒1.36) | ‒1.77 (‒2.10, ‒1.44) | ‒1.24 (‒1.54, ‒0.94) |
| LS mean difference vs. placebo (SE) | ‒0.42 (0.199) | ‒0.53 (0.206) | NA |
| *P* value | 0.03 | 0.01 |  |
| **CGIC (Week 12)** | | | |
| LS mean score (95% CI) | 0.38 (0.19, 0.57) | 0.01 (‒0.20, 0.22) | 0.46 (0.27, 0.65) |
| LS mean difference vs. placebo (SE) | ‒0.08 (0.123) | ‒0.45 (0.127) | NA |
| *P* Value | 0.51 | <0.001 |  |

^a^Per-protocol population defined as all patients who completed study treatment and had no significant protocol violations.

Abbreviations: CGIC = Clinical Global Impression of Change; CI = confidence interval; ER = extended-release; LS = least squares; MMRM = mixed model with repeated measures; NA = not applicable; SE = standard error; TNmAS-MAL = Total Numeric-modified Ashworth Scale-Most Affected Limb

**Supplementary Table 3. MMRM analysis of TNmAS-MAL and CGIC scores with pattern mixture model^a^**

|  | Arbaclofen ER  40 mg/day  (n=179) | Arbaclofen ER  80 mg/day  (n=179) | Placebo  (n=178) |
| --- | --- | --- | --- |
| **TNmAS-MAL score (Week 12)** | | | |
| LS mean change from baseline (95% CI) | ‒1.58 (‒1.89, ‒1.27) | ‒1.71 (‒2.05, ‒1.37) | ‒1.28 (‒1.57, ‒0.98) |
| LS mean difference vs. placebo (SE) | ‒0.31 (0.200) | ‒0.43 (0.207) | NA |
| *P* value | 0.12 | <0.05 |  |
| **CGIC (Week 12)** | | | |
| LS mean score (95% CI) | 0.55 (0.37, 0.74) | 0.33 (0.12, 0.54) | 0.55 (0.38, 0.73) |
| LS mean difference vs. placebo (SE) | ‒0.00 (0.119) | ‒0.22 (0.125) | NA |
| *P* value | 0.99 | 0.07 |  |

^a^Analysis performed according to the procedure described by Ratitch and O’Kelly (2011).^2^

Abbreviations: CGIC = Clinical Global Impression of Change; CI = confidence interval; ER = extended-release; LS = least squares; MMRM = mixed model with repeated measures; NA = not applicable; SE = standard error; TNmAS-MAL = Total Numeric-modified Ashworth Scale-Most Affected Limb

**Supplementary Table 4. Adverse events leading to treatment discontinuation**

| No. (%) | Arbaclofen ER  40 mg/day  (n=179) | Arbaclofen ER  80 mg/day  (n=179) | Placebo  (n=178) |
| --- | --- | --- | --- |
| Patients with adverse event leading to treatment discontinuation | 22 (12.3) | 57 (31.8) | 11 (6.2) |
| Blood and lymphatic system disorders | 0 | 0 | 0 |
| Anemia | 0 | 0 | 0 |
| Cardiac disorders | 0 | 0 | 0 |
| Acute myocardial infarction | 0 | 0 | 0 |
| Myocardial infarction | 0 | 0 | 0 |
| Ear and labyrinth disorders | 1 (0.6) | 5 (2.8) | 0 |
| Vertigo | 1 (0.6) | 5 (2.8) | 0 |
| Eye disorders | 0 | 1 (0.6) | 0 |
| Diplopia | 0 | 1 (0.6) | 0 |
| Gastrointestinal disorders | 4 (2.2) | 20 (11.2) | 0 |
| Abdominal pain upper | 0 | 1 (0.6) | 0 |
| Diarrhea | 0 | 1 (0.6) | 0 |
| Nausea | 3 (1.7) | 13 (7.3) | 0 |
| Pancreatitis, chronic | 0 | 0 | 0 |
| Paresthesia oral | 0 | 1 (0.6) | 0 |
| Vomiting | 1 (0.6) | 10 (5.6) | 0 |
| General disorders and administration site conditions | 4 (2.2) | 20 (11.2) | 4 (2.2) |
| Asthenia | 4 (2.2) | 13 (7.3) | 3 (1.7) |
| Fatigue | 0 | 1 (0.6) | 1 (0.6) |
| Gait disturbance | 0 | 10 (5.6) | 0 |
| Peripheral swelling | 0 | 1 (0.6) | 0 |

| No. (%) | Arbaclofen ER  40 mg/day  (n=179) | Arbaclofen ER  80 mg/day  (n=179) | Placebo  (n=178) |
| --- | --- | --- | --- |
| Injury, poisoning, and procedural complications | 2 (1.1) | 1 (0.6) | 1 (0.6) |
| Contusion | 1 (0.6) | 0 | 0 |
| Fall | 1 (0.6) | 0 | 0 |
| Joint injury | 0 | 1 (0.6) | 0 |
| Tibia fracture | 0 | 0 | 1 (0.6) |
| Toxicity to various agents | 0 | 0 | 0 |
| Metabolism and nutrition disorders | 0 | 1 (0.6) | 0 |
| Decreased appetite | 0 | 1 (0.6) | 0 |
| Musculoskeletal and connective tissue disorders | 8 (4.5) | 17 (9.5) | 2 (1.1) |
| Arthralgia | 1 (0.6) | 0 | 1 (0.6) |
| Muscle fatigue | 0 | 1 (0.6) | 0 |
| Muscle spasms | 0 | 1 (0.6) | 0 |
| Muscular weakness | 6 (3.4) | 15 (8.4) | 1 (0.6) |
| Myalgia | 1 (0.6) | 1 (0.6) | 0 |
| Pain in extremity | 0 | 0 | 0 |
| Neoplasms benign, malignant, and unspecified | 0 | 0 | 0 |
| Adenocarcinoma of colon | 0 | 0 | 0 |
| Meningioma | 0 | 0 | 0 |

| No. (%) | Arbaclofen ER  40 mg/day  (n=179) | Arbaclofen ER  80 mg/day  (n=179) | Placebo  (n=178) |
| --- | --- | --- | --- |
| Nervous system disorders | 10 (5.6) | 32 (17.9) | 3 (1.7) |
| Ataxia | 0 | 2 (1.1) | 0 |
| Depressed level of consciousness | 0 | 1 (0.6) | 0 |
| Disturbance in attention | 2 (1.1) | 0 | 0 |
| Dizziness | 2 (1.1) | 12 (6.7) | 0 |
| Dysarthria | 0 | 2 (1.1) | 0 |
| Headache | 0 | 1 (0.6) | 0 |
| Head discomfort | 0 | 2 (1.1) | 0 |
| Hypoesthesia | 0 | 1 (0.6) | 0 |
| Hypotonia | 0 | 4 (2.2) | 2 (1.1) |
| Loss of consciousness | 0 | 1 (0.6) | 0 |
| Multiple sclerosis relapse | 1 (0.6) | 2 (1.1) | 0 |
| Muscle spasticity | 1 (0.6) | 0 | 0 |
| Paresthesia | 0 | 0 | 1 (0.6) |
| Sedation | 0 | 1 (0.6) | 0 |
| Somnolence | 4 (2.2) | 8 (4.5) | 0 |
| Status epilepticus | 1 (0.6) | 0 | 0 |
| Psychiatric disorders | 4 (2.2) | 3 (1.7) | 1 (0.6) |
| Anxiety | 0 | 0 | 1 (0.6) |
| Anxiety disorder | 1 (0.6) | 0 | 0 |
| Confusional state | 0 | 0 | 0 |
| Delirium | 1 (0.6) | 0 | 0 |
| Depression suicidal | 0 | 1 (0.6) | 0 |
| Enuresis | 1 (0.6) | 0 | 0 |
| Hallucination | 1 (0.6) | 0 | 0 |
| Mental fatigue | 0 | 1 (0.6) | 0 |
| Panic attack | 0 | 1 (0.6) | 0 |

| No. (%) | Arbaclofen ER  40 mg/day  (n=179) | Arbaclofen ER  80 mg/day  (n=179) | Placebo  (n=178) |
| --- | --- | --- | --- |
| Renal and urinary disorders | 5 (2.8) | 9 (5.0) | 2 (1.1) |
| Atonic urinary bladder | 0 | 0 | 0 |
| Dysuria | 1 (0.6) | 0 | 0 |
| Hypertonic bladder | 0 | 0 | 1 (0.6) |
| Micturition urgency | 0 | 1 (0.6) | 0 |
| Nocturia | 0 | 1 (0.6) | 0 |
| Pollakiuria | 0 | 1 (0.6) | 0 |
| Urinary incontinence | 1 (0.6) | 4 (2.2) | 0 |
| Urinary retention | 1 (0.6) | 0 | 1 (0.6) |
| Urinary tract disorder | 4 (2.2) | 6 (3.4) | 0 |
| Reproductive system and breast disorders | 0 | 2 (1.1) | 0 |
| Erectile dysfunction | 0 | 1 (0.6) | 0 |
| Sexual dysfunction | 0 | 1 (0.6) | 0 |
| Respiratory, thoracic, and mediastinal disorders | 1 (0.6) | 0 | 0 |
| Pulmonary embolism | 1 (0.6) | 0 | 0 |
| Skin and subcutaneous tissue disorders | 1 (0.6) | 0 | 0 |
| Alopecia | 0 | 0 | 0 |
| Dermatitis allergic | 0 | 0 | 0 |
| Toxic skin eruption | 0 | 0 | 0 |
| Urticaria | 1 (0.6) | 0 | 0 |

Abbreviations: ER = extended-release

**Supplementary Table 5. USP^©^ Questionnaire scores^a^**

| Parameter | Arbaclofen ER  40 mg/day  (n=179) | Arbaclofen  ER 80 mg/day  (n=179) | Placebo  (n=178) |
| --- | --- | --- | --- |
| **Stress urinary incontinence score** | | | |
| Mean (95% CI) | 1.2 (0.8, 1.6) | 1.3 (0.9, 1.6) | 1.4 (1.1, 1.8) |
| Mean (95% CI) change from baseline | –0.2 (–0.5, 0.0) | –0.1 (–0.5, 0.3) | –0.1 (–0.4, 0.1) |
| **Overactive bladder score** | | | |
| Mean (95% CI) | 5.1 (4.4, 5.9) | 5.3 (4.6, 6.1) | 5.2 (4.6, 5.9) |
| Mean (95% CI) change from baseline | –0.8 (–1.2, –0.3) | –0.2 (–0.7, 0.4) | –0.8 (–1.3, –0.4) |
| **Low stream score** | | | |
| Mean (95% CI) | 1.6 (1.3, 1.8) | 1.4 (1.1, 1.7) | 1.5 (1.3, 1.7) |
| Mean (95% CI) change from baseline | –0.1 (–0.2, 0.1) | –0.2 (–0.4, 0.0) | –0.1 (–0.3, 0.1) |

^a^Assessed at the final study visit (Week 13).

Abbreviations: CI = confidence interval; ER = extended-release; USP = Urinary Symptom Profile

**References**

1. Polman CH, Reingold SC, Banwell B, et al. Diagnostic criteria for multiple sclerosis: 2010 revisions to the McDonald criteria. *Ann Neurol*. 2011;69(2):292–302.
2. Ratitch B, O’Kelly M. Implementation of Pattern-Mixture Models Using Standard SAS/STAT Procedures. Presented at: PharmaSUG 2011 Annual Conference; May 8-11, 2011; Nashville, TN. Paper SP04.
